# MiR-18a-5p Targets Connective Tissue Growth Factor Expression and Inhibits Transforming Growth Factor β2-Induced Trabecular Meshwork Cell Contractility

**DOI:** 10.3390/genes13081500

**Published:** 2022-08-22

**Authors:** John Knox, George Bou-Gharios, Kevin J. Hamill, Colin E. Willoughby

**Affiliations:** 1 Department of Eye and Vision Science, Institute of Life Course and Medical Sciences, University of Liverpool, Liverpool L7 8TX, UK; 2 Department of Musculoskeletal and Aging Science, Institute of Life Course and Medical Sciences, University of Liverpool, Liverpool L7 8TX, UK; 3 Genomic Medicine, Biomedical Sciences Research Institute, Ulster University, Coleraine Campus, Coleraine BT52 1SA, UK

**Keywords:** glaucoma, primary open-angle glaucoma, microRNAs, connective tissue growth factor (CTGF), TGFβ, intraocular pressure, trabecular meshwork, therapeutics

## Abstract

Increased trabecular meshwork (TM) cell and tissue contractility is a driver of the reduced outflow facility and elevation of intraocular pressure (IOP) associated with primary open-angle glaucoma (POAG). Connective tissue growth factor (CTGF) is an established mediator of TM cell contractility, and its expression is increased in POAG due to transforming growth factor β 2 (TGFβ2) signalling. Inhibiting CTGF upregulation using microRNA (miRNA) mimetics could represent a new treatment option for POAG. A combination of in silico predictive tools and a literature review identified a panel of putative CTGF-targeting miRNAs. Treatment of primary human TM cells with 5 ng/mL TGFβ2 for 24 h identified miR-18a-5p as a consistent responder, being upregulated in cells from five different human donors. Transfection of primary donor TM cells with 20 nM synthetic miR-18a-5p mimic reduced TGFβ2-induced CTGF protein expression, and stable lentiviral-mediated overexpression of this miRNA reduced TGFβ2-induced contraction of collagen gels. Together, these findings identify miR-18a-5p as a mediator of the TGFβ2 response and a candidate therapeutic agent for glaucoma via its ability to inhibit CTGF-associated increased TM contractility.

## 1. Introduction

Glaucoma, one of the leading causes of blindness worldwide [1], is characterised by degenerative optic neuropathy and progressive loss of vision [2]. Primary open-angle glaucoma (POAG) is the most prevalent subtype of glaucoma, and worldwide cases are predicted to reach 112 million by 2040 [2]. Although glaucoma pathogenesis is multifactorial [3], intraocular pressure (IOP) is the only modifiable risk factor [4]. In POAG, elevated IOP is caused by increased outflow resistance in the conventional outflow pathway [5]. One mechanism for this increased outflow resistance is increased TM contractility [6,7], which is related to the activity of TM cells, a fibroblast-like cell type that detects IOP changes as the mechanical stretch or distortion of the extracellular matrix (ECM), and results in remodelling of the actin cytoskeleton [8]. Identifying a method to reduce TM contractility by targeting the molecular or protein regulators of the system represents an option for therapeutic development in POAG.

Elevated IOP results from cellular and molecular changes in the trabecular meshwork (TM) driven by increased levels of transforming growth factor β (TGFβ), particularly TGFβ2 in the aqueous humour [9,10,11,12,13]. TGFβ2 elicits a multitude of downstream profibrotic effects, including altered turnover of ECM components, increased TM contractility, formation of cross-linked actin networks (CLANS), upregulation of α smooth muscle actin (αSMA) and aberrant formation of actin stress fibres [6,7,9,10,11,12]. However, at the centre of the TM contractility response is the matricellular protein connective tissue growth factor (CTGF) [14]. CTGF expression is increased in the TM of glaucoma patients [15] and increases TM cell contractility by driving the activation of RhoA and the formation of actin stress fibres [14]. CTGF is also implicated in fibrosis and aberrant ECM deposition in experimental and clinically significant fibrotic disorders [16]. CTGF alone can increase fibrosis and ECM deposition, showing autocrine effects to amplify its own production in the TM [17] and other disease contexts [16]. Lens-specific overexpression of CTGF in transgenic mice elevates IOP and is associated with the modification of the TM actin cytoskeleton [14] and TM cell contractility. Therefore, targeting CTGF represents an opportunity to reduce the contractility response in trabecular meshwork cells.

There are many potential approaches to target CTGF as a therapeutic anti-fibrotic strategy, including antibodies, synthetic peptides, siRNAs and CRISPR-Cas9 [18,19]. Pre-treatment with a human anti-CTGF monoclonal antibody (FG-3109; FibroGen, Inc., San Francisco, CA, USA) blocked ECM production induced with aqueous humour from glaucomatous donors in primary human TM cells isolated from glaucoma and normal subjects [20]. RNA interference (RNAi) using shRNA targeting CTGF attenuated the actin cytoskeleton in TM cells [14]. However, siRNAs directly induce mRNA cleavage; overly efficient knockdown could be problematic and also result in off-target effects [12]. CTGF is a necessary protein in the TM that increases TM cell viability in response to stress [11], and a decline in TM cellularity is seen in POAG [21]. Furthermore, siRNA efficacy is also dependent upon the region of the mRNA to which it is complementary; the need for full complementarity while avoiding off-target effects can make design challenging [13].

Therefore, we investigated the possibility of using microRNAs (miRNAs) [22] to target CTGF expression as a therapeutic strategy in glaucoma. Gene silencing is induced by miRNAs through multiple approaches, including translational repression and degradation by deadenylation, decapping or exonuclease action upon target mRNAs [22]. The effects of miRNA are generally not as potent as those induced by siRNAs, as the vast majority of targets are only partially complementary and are unable to induce endonucleolytic cleavage [22]. However, this sub-maximal response can be beneficial, as it allows miRNAs to fine-tune rather than knock out gene expression. miRNAs are also pleiotropic; their target sequences, the seed regions, frequently exist on multiple genes, and, similarly, a single seed region within a gene can be targeted by multiple different miRNAs [23]. Often, these features exist as a result of convergent evolution, with multiple genes within a pathway often sharing the same seed regions and, therefore, miRNA regulators [24,25]. In light of this important point, the manipulation of one or more miRNAs that regulate CTGF may be particularly attractive, as it could concomitantly regulate its upstream inducers and/or downstream mediators.

Several CTGF-targeting miRNAs have been discovered in different tissues and disease contexts. These include miR: 26a-5p and 26b-5p [26,27], 133b [28,29], 30c-5p [28], 218 [30], 199a-5p [31], 145-5p [32], 205-5p [33], 143-3p [34] and members of the miR-17-92 cluster: 18a-5p, 19a-3p and 19b-3p [26,35,36]. Most of these miRNAs have been reported as being expressed in the aqueous humour (AH) and TM [37,38,39], and TGFβ is known to affect the expression of some of these miRNAs [40]. In this study, we determined the effects of TGFβ2 on the expression of CTGF-targeting miRNAs in TM cells, identifying miR-18a-5p as a consistently upregulated miRNA. We then used miR-18a-5p mimics to target CTGF expression and identified that this treatment was sufficient to reduce TGFβ-induced TM cell contractility. The results suggest that a therapy based upon miR-18a-5p may hold potential for POAG treatment.

## 2. Materials and Methods

### 2.1. Human Donor Anterior Segment Collection

Corneal rim tissue, surplus tissue from corneal transplant surgery, was collected from St Paul’s Eye Unit, Royal Liverpool University Hospital, UK (Ethics Code: RETH000833). All tissue was handled in accordance with the tenets of the Declaration of Helsinki. Donor demographics are given in Supplemental Table S1.

### 2.2. Isolation, Culture, and Characterisation of Primary Human Trabecular Meshwork Cells

TM tissue was isolated by blunt dissection [41] and cut into 5–10 mm long segments, and each segment was explanted into a well of a 6-well plate (Corning Inc., Coring, NY, USA) in Dulbecco’s Modified Eagle medium (DMEM; Sigma-Aldrich, St, Louis, MO, USA) with L-glutamine (Sigma-Aldrich), supplemented with 10% foetal calf serum (FCS; Labtech, Heathfield, UK), 100 units/mL penicillin/100 µg/mL streptomycin (Sigma-Aldrich) and 2.5 µg/mL amphotericin B (Fungizone; Sigma-Aldrich). Samples were incubated in a 37 °C humidified atmosphere of 95% air and 5% CO_2_ (MCO-18AC, PHCbi, Wood Dale, IL, USA), with a medium change every 3 or 4 days. Primary human TM cells were incubated in low-glucose DMEM with L-glutamine (Sigma-Aldrich), supplemented with 10% foetal calf serum, in a 37 °C humidified atmosphere of 95% air and 5% CO_2_. All experiments were performed using TM cells under 7 passages.

Primary human TM characterisation (Figures S1, S2 and S6) was carried out as previously described [42,43] and in keeping with the published consensus [41].

For dexamethasone-mediated myocilin induction assessment, 50,000 primary TM cells were seeded onto glass coverslips in a 6-well plate (Corning Inc.) in serum-free culture medium, allowed to attach for 24 h and then treated with 100 nM dexamethasone for 24 h. Coverslips were fixed in 10% neutral buffered formalin for 5 min, washed twice with PBS, permeabilised in PBS/0.5% Triton X 100 for 5 min and then washed in 3 changes of PBS for 15 min. Coverslips were blocked using PBS/5% goat serum blocking buffer for 1.5 h at RT, washed with PBS and then incubated for 1 h at RT or overnight at 4 °C with mouse monoclonal antibodies against myocilin (3.3 µg/mL; ab55477, Abcam, Cambridge, UK) diluted in blocking buffer. Coverslips were washed and then incubated with AlexaFluor^TM^ 488 goat anti-mouse polyclonal antibodies (10 µL/mL; Thermo Fisher Scientific, Waltham, MA, USA) diluted in blocking buffer for 1 h, washed, then mounted using DAPI-containing VectaShield^®^ Antifade Mounting Medium (Vector Laboratories, Burlingame, CA, USA) and imaged using a Zeiss LSM800 Confocal Microscope (Carl Zeiss AG, Oberkochen, Germany).

For Western blotting analysis, 500,000 primary TM cells were seeded into 60 mm dishes (Corning Inc.) in serum-free culture medium, allowed to attach for 24 h and then treated or not treated with 100 nM dexamethasone in serum-free media. Cells were maintained for 7 days, with dexamethasone being replaced every day. After 7 days, dishes were washed with PBS and lysed by scraping in urea/sodium dodecyl sulphate (SDS) lysis buffer consisting of 6 M urea, 10 mM Tris-HCL (pH = 6.8), 10% glycerol, 1% SDS and 7.4 µM bromophenol blue (all Sigma-Aldrich). Then, 10% β-mercaptoethanol (final volume) was added to each lysate. Proteins were separated on a 10% SDS–polyacrylamide gel electrophoresis (PAGE) gel and then transferred onto a nitrocellulose membrane using the semi-dry Trans-Blot^®^ Turbo™ Transfer System (Bio-Rad Laboratories, Hercules, CA, USA) at 25 V (1.3 A) for 7 min. Membranes were blocked for 1 h at room temperature in Odyssey^®^ TBS-Blocking Buffer (Li-Cor Biosciences, Lincoln, NE, USA) and then probed with mouse monoclonal antibodies against myocilin (0.33 µg/mL; ab55477, Abcam) diluted in blocking buffer for either 1 h at room temperature or at 4 °C overnight. Membranes were then washed in 3 changes of PBS/0.1% Tween-20 (PBS-T) for 15 min and probed for 1 h at room temperature with IRDye^®^ 800CW-conjugated goat anti-mouse secondary antibodies (0.05 µg/mL; Li-Cor) diluted in blocking buffer. Membranes were washed with PBS-T as before, and protein bands were imaged using an Odyssey^®^ CLx 9120 Infrared Imaging System (Li-Cor).

### 2.3. In Silico miRNA Prediction and Validation

In silico analysis of miRNAs that target CTGF was performed using five independent prediction and validation tools: TargetScan [44] (http://www.targetscan.org/vert_72/; accessed on 24 January 2017), miRDB [45,46] (http://mirdb.org/; accessed on 24 January 2017), DIANA microT-CDS [47,48] (http://diana.imis.athena-innovation.gr/DianaTools/index.php?r=microT_CDS/index; accessed on 24 January 2017), miRANDA-mirSVR [49,50,51] (http://www.microrna.org/; accessed on 24 January 2017) and miRTarBase [52] (http://mirtarbase.cuhk.edu.cn/php/index.php; accessed on 24 January 2017). TargetScan ranks miRNAs that are predicted to target conserved 6-mer, 7-mer, or 8-mer that match known seed regions by their predicted efficiency of targeting (weighted context ++ score, WCS); a WCS of <0 and probability of conserved targeting (P_CT_) ≥ 0.1 were used as thresholds. miRDB scores predicted miRNA–target interactions from 0-100 based on statistical confidence; interactions with a target score (TS) ≥ 50 were selected. DIANA microT-CDS generates a combined prediction score (miTG score) for each miRNA–target interaction: the higher the score, the higher the probability of targeting; a miTG score threshold of 0.6 was applied. miRANDA-mirSVR provides a mirSVR score (SVR) of the miRNA effect on target expression and a PhastCons score (PC) of miRNA target site conservation; miRNAs with an SVR ≤ −0.1 and a PC > 0.57 were selected. miRTarBase is a database of miRNA–target interactions and displays miRNAs validated to target genes by a range of methods, with links to the relevant literature demonstrating the validation. A review of the literature was also conducted using PubMed (https://pubmed.ncbi.nlm.nih.gov/; accessed 24 on January 2017) and the search terms “CTGF” and “miRNA”. A second literature review investigated whether any candidate miRNAs are expressed in the TM or AH.

### 2.4. Treatment of Primary Human TM Cells with TGFβ2 for miRNA Expression Measurement

Primary human TM cells in T75 flasks were grown to 70–80% confluence. Flasks were then serum-starved for 24 h and then either treated with 5 ng/mL TGFβ2 (R&D Systems, Minneapolis, MN, USA) or maintained in serum-free medium for 24 h. Cells were then trypsinised and resuspended in RTL lysis buffer (Qiagen, Hilden, Germany) for RNA extraction.

### 2.5. miRNA and mRNA RT-qPCR

Total RNA was extracted using AllPrep^®^ DNA/RNA/miRNA Universal Kit (Qiagen) as per the manufacturer’s specifications. RNA quality was measured by UV spectrophotometry using a Nanodrop 2000 (Thermo Fisher Scientific); RNAs with a minimum 260/280 ratio of 1.90 and a minimum concentration of 10 ng/µL were used. For miRNA analyses, 1 µg of cDNA was synthesised using miScript II RT kit (Qiagen) and miScript SYBR^®^ Green PCR mix with miScript universal primer (miScript primer assays, Qiagen) (Table S2) used for quantitative PCR. SNORD61 and SNORD68 were used as reference transcripts. For mRNA analyses, Precision™ nanoScript 2 Reverse Transcription kit (Primerdesign, Southampton, UK) was used to generate 100 ng of cDNA, as per the manufacturer’s specifications, and PrecisionPLUS SYBR q-PCR Master Mix (Primerdesign) was used to measure mRNA expression (Table S3). RPLP0 and GAPDH were used as reference transcripts. Quantitative PCR was performed using a LightCycler 96 (Roche Holding AG, Basel, Switzerland). Relative fold changes were analysed using the comparative CT (ΔΔCt [53]) method using the geometric mean of the reference transcripts.

### 2.6. miRNA and siRNA Transfection

Primary human TM cells were seeded at 100,000 cells per well in a 6-well plate in serum-free medium. After 24 h, TM cells were treated with 5 ng/mL TGFβ2 and transfected with 20 nM synthetic human miRNA mimic for miR-18a, 20 nM miRNA inhibitor for miR-18a, 20 nM CTGF siRNA or a scrambled negative control (Table 1). The transfection mix was prepared using 15 µL of HiPerfect Transfection reagent (301704, Qiagen) per transfection and allowed to mix for 15 min before being applied to the cells. Media were removed after 24 h for RNA experiments or 72 h for protein experiments, and all wells were washed with PBS before the cells were lysed by scraping in Buffer RTL for RNA or urea/SDS lysis buffer for protein analyses. For SDS-PAGE and Western blotting, protein samples were processed as described for cell characterisation but using rabbit polyclonal antibodies against CTGF (1 µg/mL; ab6992, Abcam) and rabbit monoclonal antibodies against lamin A (0.183 µg/mL; ab108922 [EPR4068], Abcam) with IRDye^®^ 680RD-conjugated goat anti-rabbit secondary antibodies (0.05 µg/mL; Li-Cor). Furthermore, blots for lamin A were transferred onto a nitrocellulose membrane by wet transfer at 100 V for 1 h.

### 2.7. shRNA Transduction, Selection and Experimentation

Primary human TM cells at passage 3 were plated at 100,000 cells per well in 6-well plates and allowed to attach overnight. Cells were transduced with lentiviruses (GE Healthcare Dharmacon, Inc., Lafayette, CO, USA) carrying shMIR-18a, shCTGF or a non-targeting control (NTC) at a multiplicity of infection of 1, with 12 µg/mL polybrene. The medium was replaced after 24 h, and cells were maintained in growth medium for one week. Cells were then selected using 1 µg/mL puromycin (Gibco, Thermo Fisher Scientific) for one week. Selected cells were then imaged using a Nikon Eclipse Ti microscope (Nikon, Minato-ku, Tokyo, Japan) for fluorescent markers: red fluorescent protein (RFP) in NTC and miR-18a shMimic-transduced cells and green fluorescent protein (GFP) in shCTGF-transduced cells.

### 2.8. Collagen Gel Contraction Assay

Primary human TM cells were trypsinised and resuspended in culture medium in 1.2 mL of a gel mix of 10× PBS, rat tail collagen 1 (Corning Inc.), cell suspension and NaOH (Sigma-Aldrich) for a final collagen concentration of 1.5 mg/mL and a final cell concentration of 4 × 10^5^ cells/mL. Then, 500 µL of each gel mix was pipetted into 2 wells of a 24-well plate (Corning Inc.), and the gels were allowed to set for 45 min in a 37 °C humidified incubator with 5% CO_2_. Once the gels were set, 500 µL of growth medium was added, and the gels were incubated for a further 48 h. The gels were then serum-starved for 24 h before one gel of each transduced cell type was treated with 5 ng/mL TGFβ2. The gels were then detached from the bottom of the wells using a 10 µL pipette tip and imaged at 0, 24 and 48 h after treatment using a ChemiDoc XRS+ system (Bio-Rad Laboratories).

### 2.9. Statistical Analysis

Statistical analyses were performed using GraphPad software (GraphPad Software, San Diego, CA, USA). Student’s *t*-test with Holm–Sidak correction for multiple comparisons was used to analyse miRNA qPCR data. The Friedman test with Dunn’s multiple comparisons test was used to analyse mRNA and Western blot data.

## 3. Results

### 3.1. In Silico Identification of CTGF-Targeting miRNAs

In silico prediction and miRNA alignment tools were used to identify candidate miRNAs with the potential to regulate CTGF. Outputs from TargetScanHuman (http://www.targetscan.org/vert_72/; accessed on 24 January 2017), miRDB (http://mirdb.org/; accessed on 24 January 2017), miRANDA-mirSVR (http://www.microrna.org/; accessed on 24 January 2017) and DIANA-microT-CDS (http://diana.imis.athena-innovation.gr/DianaTools/index.php?r=microT_CDS/index; accessed on 24 January 2017) were compared, and candidate miRNAs were chosen if they reached the detection threshold in three or more of the predictive tools. In total, 18 miRNAs met these criteria (Table 2), and of these, 10 were reported to be validated according to the miRTarBase database (http://mirtarbase.cuhk.edu.cn/php/index.php; accessed on 24 January 2017).

In addition, a review of the literature was performed using NCBI–PubMed to investigate miRNAs that target CTGF and to identify whether any of the identified candidates had been detected in the TM and/or AH. These reviews further narrowed the list of miRNAs to investigate to eight: miR-133b, miR-18a-5p, miR-199a-5p, miR-199b-5p, miR-19a-3p, miR-19b-3p, miR-26a-5p and miR-26b-5p.

### 3.2. Expression of miR-18a-5p Increases in Primary Human TM Cells after TGFβ2 Treatment

**Figure 1:**
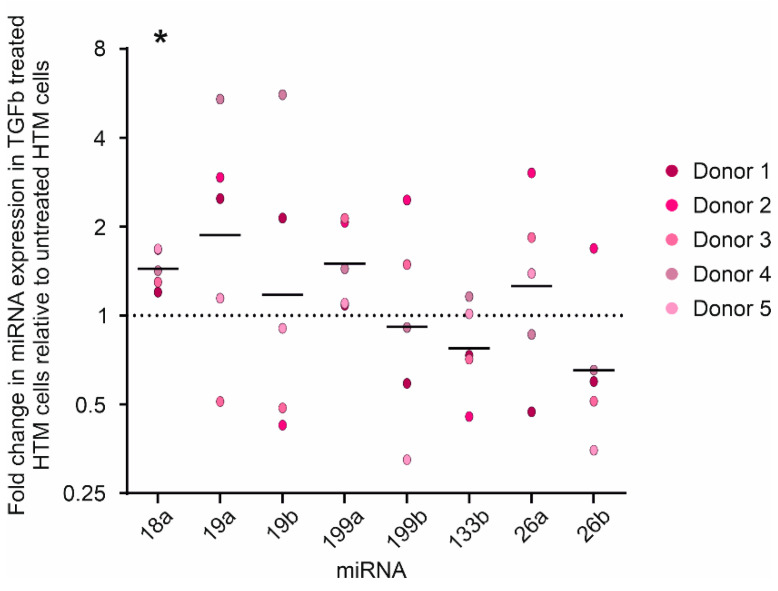
**miR-18a-5p expression increases in human TM cells treated with TGFβ2.** Fold change in miRNA expression relative to untreated human TM cells, normalised to the geometric mean of SNORD61 and SNORD68. Individual values for donors 1 to 5 are shown, with the mean ΔΔCt shown as a bar. The line at y = 1 represents the level of expression in untreated cells. * *p* = 0.0006, *n* = 5, Student’s *t*-test with Holm–Sidak correction for multiple comparisons.

The expression of the eight candidate miRNAs was measured in primary human TM cells with and without treatment with 5 ng/mL TGFβ2 for 24 h. Analyses revealed that miR-18a-5p showed consistent upregulation in response to TGFβ2 in human TM cells relative to untreated cells in all five donor cell lines (1.45 ± 0.22 fold, *p* = 0.0006; Figure 1). The other miRNAs showed donor variability, and no other miRNAs displayed a consistent response to TGFβ2 across the entire cohort. However, the expression of miR-19a, miR-199a and miR-26a displayed a general tendency toward increased expression, while the expression of miR-133b and miR-26b tended to decrease in response to TGFβ2 treatment.

### 3.3. miR-18a-5p Mimic Reduces TGFβ2-Induced CTGF mRNA and Protein Upregulation in Human TM Cells

**Figure 2:**
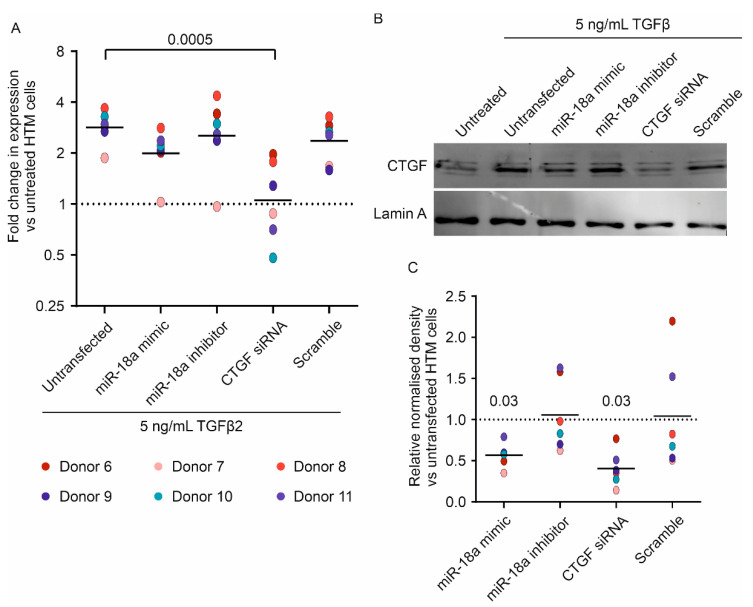
**miR-18a-5p mimic and CTGF siRNA reduce CTGF expression.** (**A**) CTGF expression was measured by RT-qPCR, relative to untreated cells, and normalised to the geomean of RPLP0 and GAPDH. Individual values for donors 6 to 11 are shown with a bar representing the median. The line at y = 1 represents the level of expression in untreated cells. Data were analysed by the Friedman test with Dunn’s multiple comparisons test, *n* = 6. (**B**) Representative Western blots of CTGF and lamin A. (**C**) Densitometry analysis of CTGF blots, normalised to lamin A. Individual values are shown with a bar representing the median. The line at y = 1 represents the level of TGFβ2-treated cells. Data were analysed by Wilcoxon signed-rank test comparing median values to a hypothetical value of 1, *n* = 6.

Next, transient transfection with a miR-18a-5p mimic was used to determine if increasing the expression of this miRNA affected CTGF expression in TGFβ2-treated primary human TM cells (Figure 2). A CTGF-targeting siRNA was used as a positive control, and a scrambled siRNA was used as a negative control. mRNA expression was measured after 24 h, and protein expression was measured after 72 h. TGFβ2 treatment resulted in a 2.9-fold increase in CTGF expression (range: 1.9–3.7), which was almost completely inhibited by CTGF siRNA (median 1.1, range: 0.5–2.0, *p* = 0.0005 vs. TGFβ2-treated human TM cells; Figure 2A). In all donors, transfection of the miR-18a mimic reduced the increase in CTGF caused by TGFβ2, although this did not reach statistical significance at a significance level of 0.05 (median 2.2, range: 1.0–2.8, *p* = 0.07 vs. TGFβ2-treated human TM cells).

At the protein level, there was donor-to-donor variability in the baseline level of CTGF and also in the extent of the increase in CTGF protein expression in response to TGFβ2 treatment. However, as expected, TGFβ2 did increase CTGF protein abundance in all donors (Figure 2B, Figures S3 and S7). miR-18a-5p mimic transfection decreased the CTGF protein levels compared to TGFβ2-treated cells (miRNA mimic: median 0.58 relative to untransfected cells, range: 0.35–0.79, *p* = 0.03); this reduction was slightly less than the effect of CTGF siRNA (siRNA median 0.37, range: 0.14–0.77, *p* = 0.03; Figure 2C).

### 3.4. Lentiviral-Driven Overexpression of miR-18a-5p Leads to Reduced Human TM Cell Contractility in Response to TGFβ2

We next investigated whether induced mir-18a expression was sufficient to inhibit TGFβ2-induced TM cell contractility. Lentiviral miR-18a-5p (shMIR-18a), CTGF shRNA (shCTGF) or a non-targeting control (NTC) was used to allow for long-term expression in two different donors (8 and 9). Virally transduced human TM cells were selected through antibiotic resistance, and transduction was confirmed by the expression of RFP or GFP (Figure S4). Transduced cells were tested for their ability to respond to dexamethasone by increasing myocilin expression to confirm that the cells retained TM characteristics after selection (Figure S5).

**Figure 3:**
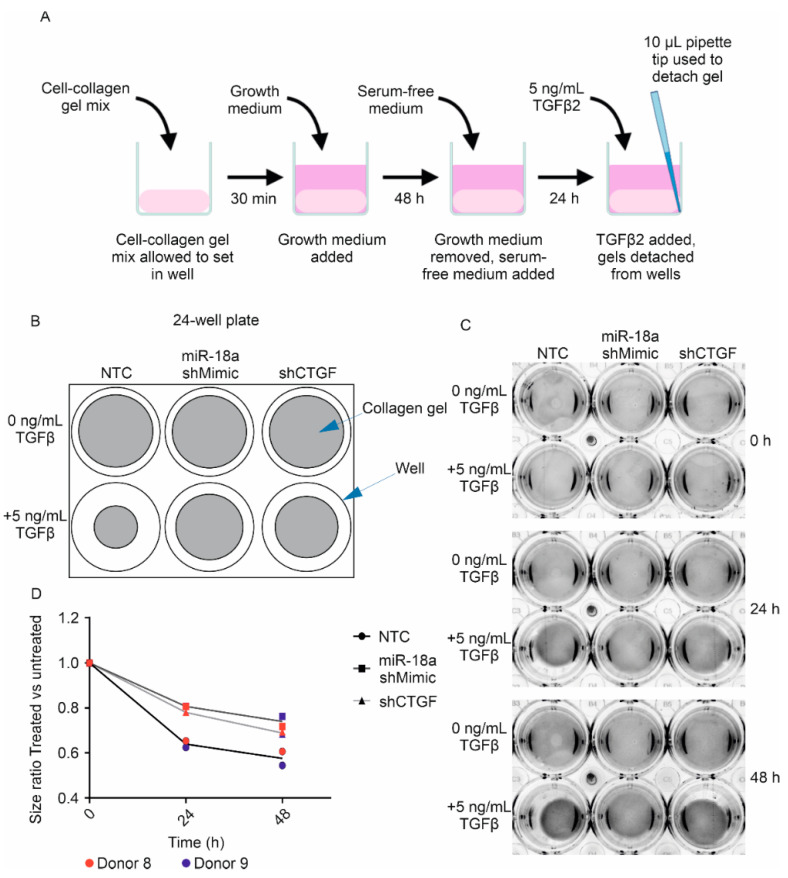
**TM cells have reduced ability to contract collagen gels when miR-18a-5p is overexpressed.** (**A**) A diagram showing the method used to make collagen gels. (**B**) Representative diagram showing the wells of a 24-well plate with collagen gels in them. (**C**) Images of collagen gels seeded with lentiviral-transduced TM cells overexpressing miR-18a, shCTGF or a non-targeting control at 0, 24 and 48 h after TGFβ2 treatment compared to untreated gels. (**D**) Ratio of the area of TGFβ2-treated collagen gels vs. untreated gels. Lines connect median size ratio for each group, *n* = 2.

NTC, shMIR-18a and shCTGF-TM cells were seeded into collagen gels and treated with 5 ng/mL TGFβ2 (Figure 3A). Images were taken at 0 h, 24 h and 48 h after TGFβ2 treatment (Figure 3C), and the area of the gels was measured and compared to the area of untreated gels at the same time point. Twenty-four hours of TGFβ2 treatment resulted in the contraction of collagen gels seeded with NTC-TM cells (donor 8: 0.62-fold, donor 9: 0.65-fold relative to untreated NTC-TM; Figure 3D). In the cells induced to express shMIR-18a, the amount of TGFβ2-induced contractility was less than in the NTC controls (shMIR-18a donor 8: 0.81-fold and donor 9: 0.81-fold). This was also the case for the cells expressing shCTGF (shCTGF donor 8: 0.78-fold and donor 9: 0.78-fold).

At 48 h TGFβ2 treatment, the collagen gels seeded with NTC-TM cells had contracted a little more relative to untreated gels (donor 8: 0.61-fold, donor 9: 0.54-fold). Again, the amount of TGFβ2 contraction was reduced in cells expressing shMIR-18a (shMIR-18a donor 8: 0.72-fold, donor 9: 0.76-fold relative to untreated gels) and in cells expressing shCTGF (shCTGF donor 8: 0.69-fold, donor 9: 0.68-fold). These results demonstrate that primary human TM cell contractility can be reduced by the overexpression of miR-18a-5p.

## 4. Discussion

Profibrotic changes elicited by TGFβ2 within the TM have been implicated in the pathogenesis of POAG [9,11]. TGFβ is a multifunctional cytokine, and global inhibition of its signalling pathway in the TM would cause considerable deleterious effects [56,57]. CTGF is highly expressed in the TM [58] and plays a pivotal role in the pathogenesis of glaucoma [14,16]. Targeting CTGF, a downstream effector protein of TGFβ-induced TM pathology, would not impact the pleiotropic effects of TGFβ and would provide a more targeted approach, limiting the risk of potential adverse events [57]. Herein, we identify the potential of miR-18a-5p as the basis of a CTGF-targeting therapy for POAG. The development of disease-modifying therapeutics targeting TGFβ in the TM to treat glaucoma based on microRNA biology could offer substantial clinical benefits.

In this study, transient transfection of miR-18a-5p mimics reduced the expression of CTGF following TGFβ2 treatment in primary human TM cells, while lentiviral-mediated expression reduced TM cell contractility in response to TGFβ2, a phenotype associated with POAG. Our interpretation of the contraction assay is limited by the small number of biological replicates and lack of statistical analysis. However, the low variability between donors within each group suggests that we are seeing a real effect of miR-18a-5p overexpression, although this experiment should be replicated with further donor cells to confirm these results. Increased TM cell contractility modifies the structure and organisation of the ECM, resulting in increased tissue contractility and stiffness. In POAG, decreasing TM cell contractility would therefore decrease TM tissue contractility and stiffness [59]. Indeed, inhibition of Rho kinase is known to reduce TM cell contractility and increase AH outflow [60]. Contraction of free-floating collagen gels is mediated, in part, by RhoA signalling [61], and knockdown of CTGF expression in TM cells has been demonstrated to reduce RhoA signalling [14]. While miR-18a-5p is not known to target members of the RhoA pathway, it does target CTGF, which can activate RhoA signalling in TM cells in an integrin-dependent manner [14]. The effectiveness of miR-18a-5p upregulation on TM cell contractility was similar to that of shRNA CTGF knockdown. Given that miR-18a-5p downregulates CTGF expression in TM cells, this suggests that CTGF downregulation is the mechanism by which miR-18a-5p inhibits TM cell contractility. Therefore, miR-18a-5p can be used as the basis of a CTGF-targeting therapy to reduce TM cell contractility in POAG.

There may be a potential clinical benefit in targeting both ROCK and CTGF in terms of IOP control. In CTGF-overexpressing transgenic mice, topical administration of Rho kinase (ROCK) inhibitors lowered IOP [14] by reducing CTGF-induced TM contractility. Netarsudil and ripasudil are ROCK inhibitors approved in the USA and Japan, respectively, for the treatment of glaucoma. CTGF-targeting therapies could work in tandem with ROCK inhibitors, as TGFβ, CTGF and RhoA/ROCK signalling interact with and synergise with each other in TM cells [14,62]. As this study has shown that miR-18a-5p overexpression reduces CTGF expression and TM cell contractility, this therapeutic strategy could work synergistically with ROCK inhibitor therapy in glaucoma. The concept of a synergistic treatment is particularly compelling, as targeting ROCK on its own would not necessarily prevent TGFβ-driven fibrosis in the TM. Further investigation into miR-18a-5p-based therapies on CTGF expression and TM contractility should include ROCK inhibition. miR-18a-5p also targets TGF-βRII [63], SMAD2 [64] and SMAD4 [64], which could also contribute to suppressing the TGFβ signalling pathway and now requires further study. The role of miR-18a in ECM regulation, EMT and cell cycle and apoptosis requires further study in cellular and in vivo models [65].

miRNA-based therapeutic CTGF targeting has broader potential in ocular and non-ocular fibrotic diseases. Beyond the trabecular meshwork, CTGF-driven fibrosis has been implicated in the lamina cribrosa [66] and optic nerve head astrocytes [67] in glaucoma and is associated with surgical scarring following glaucoma filtration surgery [68,69]. CTGF also contributes to intraocular fibrosis in diabetic retinopathy [70], age-related macular degeneration [71] and proliferative vitreoretinopathy [72].

RNA-based therapies for glaucoma offer the potential to deliver disease-modifying molecules targeting the molecular pathology of this blinding ocular condition [73]. In the study described herein, miR-18a-5p was identified as a candidate therapeutic agent for glaucoma via its ability to inhibit CTGF-associated increased TM contractility.

## Figures and Tables

**Table 1:** Sequences of miScript miR-18a mimic and inhibitor, CTGF siRNA and scrambled negative control.

Synthetic RNA	Sequence	Qiagen ID
hsa-miR-18a-5p mimic	5′-UAAGGUGCAUCUAGUGCAGAUAG	MSY0000072
hsa-miR-18a-5p inhibitor	5′-CUAUCUGCACUAGAUGCACCUUA	MIN0000072
FlexiTube CTGF siRNA	5′-UAUCUGAUGAUACUAACCUTT	SI00029680
Scrambled negative control	Proprietary; no known homology to any mammalian gene	1027280

**Table 2:** Glaucoma-relevant miRNAs predicted or validated to target CTGF.

miRNA	TargetScan	miRDB TS	miRANDA-mirSVR	MicroT-CDS miTG Score	miRTarBase Validation	Literature	TM/AH Expression
miR-124-3p	WCS: −0.38 P_CT_: 0.22	74	N/A	0.66	RA, microA		
miR-1297	WCS: −0.29 P_CT_: 0.69	65	SVR: −0.1096 PC: 0.7355	0.96	Not validated		
miR-132-3p	WCS: −0.28 P_CT_: 0.26	93	SVR: −1.2409 PC: 0.7586	0.68	Not validated		TM [38]
miR-133a-3p	WCS: −0.31 P_CT_: 0.8	56	N/A	0.91	Not validated	[54]	AH [37] TM [38]
**miR-133b**	WCS: −0.31 P_CT_: 0.8	93	SVR: −0.2487 PC: 0.7793	0.91	RA, WB, qPCR	[28,29,54]	AH [37]
miR-143-3p	WCS: −0.24 P_CT_: 0.26	76	SVR: −0.2242 PC: 0.7355	0.84	RA	[34]	AH [37] TM [38]
**miR-18a-5p**	WCS: −0.37 P_CT_: 0.45	86	SVR: −1.0860 PC: 0.7793	0.91	RA, WB, microA	[36]	TM [39]
miR-18b-5p	WCS: −0.37 P_CT_: 0.45	86	SVR: −1.0860 PC: 0.7793	0.91	RA, WB	[55]	
**miR-199a-5p**	WCS: −0.19 P_CT_: 0.24	63	SVR: −0.4603 PC: 0.6405	0.65	Not validated	[31]	AH [37] TM [38]
**miR-199b-5p**	WCS: −0.2 P_CT_: 0.24	63	SVR: −0.4603 PC: 0.6405	0.65	Not validated		AH [37] TM [38,39]
**miR-19a-3p**	WCS: −0.26 P_CT_: 0.82	No score	SVR: −0.7564 PC: 0.7793	0.75	Other ^†^	[26]	AH [37] TM [38]
**miR-19b-3p**	WCS: −0.27 P_CT_: 0.82	No score	SVR: −0.7278 PC: 0.7793	0.76	Other ^†^	[26,36]	AH [37] TM [38]
miR-212-3p	WCS: −0.3 P_CT_: 0.26	93	SVR: −1.2409 PC: 0.7586	0.70	Not validated		
**miR-26a-5p**	WCS: −0.31 P_CT_: 0.69	57	SVR: −0.1085 PC: 0.7355	0.95	WB, microA	[27]	AH [37] TM [38]
**miR-26b-5p**	WCS: −0.29 P_CT_: 0.69	57	SVR: −0.1085 PC: 0.7355	0.95	RA, WB, qPCR	[26]	AH [37] TM [38]
miR-4735-3p	WCS: −0.36 P_CT_: 0.45	86	N/A	0.89	Not validated		
miR-4770	WCS: −0.24 P_CT_: 0.26	76	N/A	0.83	Not validated		
miR-6088	WCS: −0.24 P_CT_: 0.26	72	N/A	0.62	Not validated		

^†^ miRNA effects on biological function. miRNAs in bold were selected for further study. WCS, weighted context ++ score. TS, target score. SVR, mirSVR score. PC, PhastCons score. RA, reporter assay. microA, microarray. WB, Western blot. AH, aqueous humour. TM, trabecular meshwork.

## Data Availability

Data are contained within the article.

## Supplementary Materials

The following supporting information can be downloaded at: https://www.mdpi.com/article/10.3390/genes13081500/s1. Table S1: Demographic information of human donor rims; Table S2: miScript primer assay sequences; Table S3: Unlabelled gene-specific human primers; Figure S1: Characterisation of donor TM cells; Figure S2: Characterisation of TM cells from donors 4 to 11 by Western blot for myocilin; Figure S3: All Western blots for CTGF and Lamin A; Figure S4: Validation of lentiviral cell lines; Figure S5: Lentiviral-transduced TM cells display characteristic upregulation of myocilin by dexamethasone; Figure S6: Original myocilin blot images; Figure S7: Original CTGF and Lamin A blot images.

## References

[1] Bourne R.R.A., Steinmetz J.D., Flaxman S., Briant P.S., Taylor H.R., Resnikoff S., Casson R.J., Abdoli A., Abu-Gharbieh E., Afshin A. (2021). Trends in prevalence of blindness and distance and near vision impairment over 30 years: An analysis for the Global Burden of Disease Study. Lancet Glob. Health.

[2] Jonas J.B., Aung T., Bourne R.R., Bron A.M., Ritch R., Panda-Jonas S. (2017). Glaucoma. Lancet.

[3] Alqawlaq S., Flanagan J.G., Sivak J.M. (2019). All roads lead to glaucoma: Induced retinal injury cascades contribute to a common neurodegenerative outcome. Exp. Eye Res..

[4] Wormald R., Virgili G., Azuara-Blanco A. (2019). Systematic reviews and randomised controlled trials on open angle glaucoma. Eye.

[5] Gottanka J., Johnson D.H., Martus P., Lütjen-Drecoll E. (1997). Severity of optic nerve damage in eyes with POAG is correlated with changes in the trabecular meshwork. J. Glaucoma.

[6] Lepple-Wienhues A., Stahl F., Wiederholt M. (1991). Differential smooth muscle-like contractile properties of trabecular meshwork and ciliary muscle. Exp. Eye Res..

[7] Wiederholt M. (1998). Direct involvement of trabecular meshwork in the regulation of aqueous humor outflow. Curr. Opin. Ophthalmol..

[8] Tian B., Gabelt B.T., Geiger B., Kaufman P.L. (2009). The role of the actomyosin system in regulating trabecular fluid outflow. Exp. Eye Res..

[9] Fuchshofer R., Tamm E.R. (2012). The role of TGF-β in the pathogenesis of primary open-angle glaucoma. Cell Tissue Res..

[10] Keller K.E., Aga M., Bradley J.M., Kelley M.J., Acott T.S. (2009). Extracellular matrix turnover and outflow resistance. Exp. Eye Res..

[11] Wordinger R.J., Sharma T., Clark A.F. (2014). The role of TGF-β2 and bone morphogenetic proteins in the trabecular meshwork and glaucoma. J. Ocul. Pharmacol. Ther..

[12] Stamer W.D., Acott T.S. (2012). Current Understanding of Conventional Outflow Dysfunction in Glaucoma. Curr. Opin. Ophthalmol..

[13] Prendes M.A., Harris A., Wirostko B.M., Gerber A.L., Siesky B. (2013). The role of transforming growth factor β in glaucoma and the therapeutic implications. Br. J. Ophthalmol..

[14] Junglas B., Kuespert S., Seleem A.A., Struller T., Ullmann S., Bösl M., Bosserhoff A., Köstler J., Wagner R., Tamm E.R. (2012). Connective tissue growth factor causes glaucoma by modifying the actin cytoskeleton of the trabecular meshwork. Am. J. Pathol..

[15] Browne J.G., Ho S.L., Kane R., Oliver N., Clark A.F., O’Brien C.J., Crean J.K. (2011). Connective tissue growth factor is increased in pseudoexfoliation glaucoma. Investig. Ophthalmol. Vis. Sci..

[16] Wallace D.M., Murphy-Ullrich J.E., Downs J.C., O’Brien C.J. (2014). The role of matricellular proteins in glaucoma. Matrix Biol..

[17] Junglas B., Yu A.H.L., Welge-Lüssen U., Tamm E.R., Fuchshofer R. (2009). Connective tissue growth factor induces extracellular matrix deposition in human trabecular meshwork cells. Exp. Eye Res..

[18] Chen Z., Zhang N., Chu H.Y., Yu Y., Zhang Z.K., Zhang G., Zhang B.T. (2020). Connective Tissue Growth Factor: From Molecular Understandings to Drug Discovery. Front. Cell Dev. Biol..

[19] Lee E.J., Han J.C., Park D.Y., Cho J., Kee C. (2021). Effect of connective tissue growth factor gene editing using adeno-associated virus-mediated CRISPR-Cas9 on rabbit glaucoma filtering surgery outcomes. Gene Ther..

[20] Wallace D.M., Clark A.F., Lipson K.E., Andrews D., Crean J.K., O’Brien C.J. (2013). Anti-connective tissue growth factor antibody treatment reduces Extracellular matrix production in Trabecular meshwork and Lamina Cribrosa cells. Investig. Ophthalmol. Vis. Sci..

[21] Fan X., Bilir E.K., Kingston O.A., Oldershaw R.A., Kearns V.R., Willoughby C.E., Sheridan C.M. (2021). Replacement of the Trabecular Meshwork Cells-A Way Ahead in IOP Control?. Biomolecules.

[22] Huntzinger E., Izaurralde E. (2011). Gene silencing by microRNAs: Contributions of translational repression and mRNA decay. Nat. Rev. Genet..

[23] Kehl T., Backes C., Kern F., Fehlmann T., Ludwig N., Meese E., Lenhof H.P., Keller A. (2017). About miRNAs, miRNA seeds, target genes and target pathways. Oncotarget.

[24] Kern F., Krammes L., Danz K., Diener C., Kehl T., Küchler O., Fehlmann T., Kahraman M., Rheinheimer S., Aparicio-Puerta E. (2021). Validation of human microRNA target pathways enables evaluation of target prediction tools. Nucleic Acids Res..

[25] Kehl T., Kern F., Backes C., Fehlmann T., Stöckel D., Meese E., Lenhof H.P., Keller A. (2020). MiRPathDB 2.0: A novel release of the miRNA Pathway Dictionary Database. Nucleic Acids Res..

[26] Chen Y.C., Chen B.C., Yu C.C., Lin S.H., Lin C.H. (2016). miR-19a, -19b, and -26b Mediate CTGF Expression and Pulmonary Fibroblast Differentiation. J. Cell. Physiol..

[27] Koga K., Yokoi H., Mori K., Kasahara M., Kuwabara T., Imamaki H., Ishii A., Mori K.P., Kato Y., Ohno S. (2015). MicroRNA-26a inhibits TGF-β-induced extracellular matrix protein expression in podocytes by targeting CTGF and is downregulated in diabetic nephropathy. Diabetologia.

[28] Duisters R.F., Tijsen A.J., Schroen B., Leenders J.J., Lentink V., van der Made I., Herias V., van Leeuwen R.E., Schellings M.W., Barenbrug P. (2009). miR-133 and miR-30 regulate connective tissue growth factor: Implications for a role of microRNAs in myocardial matrix remodeling. Circ. Res..

[29] Guo Y., Li X., Lin C., Zhang Y., Hu G., Zhou J., Du J., Gao K., Gan Y., Deng H. (2015). MicroRNA-133b inhibits connective tissue growth factor in colorectal cancer and correlates with the clinical stage of the disease. Mol. Med. Rep..

[30] Lun W., Wu X., Deng Q., Zhi F. (2018). MiR-218 regulates epithelial-mesenchymal transition and angiogenesis in colorectal cancer via targeting CTGF. Cancer Cell Int..

[31] Sun D., Han S., Liu C., Zhou R., Sun W., Zhang Z., Qu J. (2016). Microrna-199a-5p functions as a tumor suppressor via suppressing connective tissue growth factor (CTGF) in follicular thyroid carcinoma. Med. Sci. Monit..

[32] Lee H.K., Bier A., Cazacu S., Finniss S., Xiang C., Twito H., Poisson L.M., Mikkelsen T., Slavin S., Jacoby E. (2013). MicroRNA-145 Is Downregulated in Glial Tumors and Regulates Glioma Cell Migration by Targeting Connective Tissue Growth Factor. PLoS ONE.

[33] Xie H., Zhao Y., Caramuta S., Larsson C., Lui W.-O. (2012). miR-205 Expression Promotes Cell Proliferation and Migration of Human Cervical Cancer Cells. PLoS ONE.

[34] Mu S., Kang B., Zeng W., Sun Y., Yang F. (2016). MicroRNA-143-3p inhibits hyperplastic scar formation by targeting connective tissue growth factor CTGF/CCN2 via the Akt/mTOR pathway. Mol. Cell. Biochem..

[35] Guo Y., Lu X., Wang H. (2016). Downregulation of miR-18a induces CTGF and promotes proliferation and migration of sodium hyaluronate treated human corneal epithelial cells. Gene.

[36] van Almen G.C., Verhesen W., van Leeuwen R.E.W., van de Vrie M., Eurlings C., Schellings M.W.M., Swinnen M., Cleutjens J.P.M., van Zandvoort M.A.M.J., Heymans S. (2011). MicroRNA-18 and microRNA-19 regulate CTGF and TSP-1 expression in age-related heart failure. Aging Cell.

[37] Wecker T., Hoffmeier K., Plötner A., Grüning B.A., Horres R., Backofen R., Reinhard T., Schlunck G. (2016). MicroRNA profiling in aqueous humor of individual human eyes by next-generation sequencing. Investig. Ophthalmol. Vis. Sci..

[38] Drewry M., Helwa I., Allingham R.R., Hauser M.A., Liu Y. (2016). miRNA profile in three different normal human ocular tissues by miRNA-seq. Investig. Ophthalmol. Vis. Sci..

[39] Li G., Luna C., Qiu J., Epstein D.L., Gonzalez P. (2009). Alterations in microRNA expression in stress-induced cellular senescence. Mech. Ageing Dev..

[40] Butz H., Rácz K., Hunyady L., Patócs A. (2012). Crosstalk between TGF-β signaling and the microRNA machinery. Trends Pharm. Sci..

[41] Keller K.E., Bhattacharya S.K., Borrás T., Brunner T.M., Chansangpetch S., Clark A.F., Dismuke W.M., Du Y., Elliott M.H., Ethier C.R. (2018). Consensus recommendations for trabecular meshwork cell isolation, characterization and culture. Exp. Eye Res..

[42] Callaghan B., Lester K., Lane B., Fan X., Goljanek-Whysall K., Simpson D.A., Sheridan C., Willoughby C.E. (2022). Genome-wide transcriptome profiling of human trabecular meshwork cells treated with TGF-β2. Sci. Rep..

[43] Kathirvel K., Karen L., Haribalaganesh R., Krishnadas R., Muthukkaruppan V., Lane B., Simpson D.A., Goljanek-Whysall K., Sheridan C., Bharanidharan D. (2022). Short and long-term effect of dexamethasone on the transcriptome profile of primary human trabecular meshwork cells in vitro. Sci. Rep..

[44] Agarwal V., Bell G.W., Nam J.-W., Bartel D.P. (2015). Predicting effective microRNA target sites in mammalian mRNAs. Elife.

[45] Chen Y., Wang X. (2020). MiRDB: An online database for prediction of functional microRNA targets. Nucleic Acids Res..

[46] Liu W., Wang X. (2019). Prediction of functional microRNA targets by integrative modeling of microRNA binding and target expression data. Genome Biol..

[47] Reczko M., Maragkakis M., Alexiou P., Grosse I., Hatzigeorgiou A.G. (2012). Functional microRNA targets in protein coding sequences. Bioinformatics.

[48] Paraskevopoulou M.D., Georgakilas G., Kostoulas N., Vlachos I.S., Vergoulis T., Reczko M., Filippidis C., Dalamagas T., Hatzigeorgiou A.G. (2013). DIANA-microT web server v5.0: Service integration into miRNA functional analysis workflows. Nucleic Acids Res..

[49] Betel D., Wilson M., Gabow A., Marks D.S., Sander C. (2008). The microRNA.org resource: Targets and expression. Nucleic Acids Res..

[50] Enright A.J., John B., Gaul U., Tuschl T., Sander C., Marks D.S. (2003). MicroRNA targets in Drosophila. Genome Biol..

[51] John B., Enright A.J., Aravin A., Tuschl T., Sander C., Marks D.S. (2004). Human microRNA targets. PLoS Biol..

[52] Chou C.H., Shrestha S., Yang C.D., Chang N.W., Lin Y.L., Liao K.W., Huang W.C., Sun T.H., Tu S.J., Lee W.H. (2018). MiRTarBase update 2018: A resource for experimentally validated microRNA-target interactions. Nucleic Acids Res..

[53] Livak K.J., Schmittgen T.D. (2001). Analysis of relative gene expression data using real-time quantitative PCR and the 2^−ΔΔCT^ method. Methods.

[54] Duan L.J., Qi J., Kong X.J., Huang T., Qian X.Q., Xu D., Liang J.H., Kang J. (2015). MiR-133 modulates TGF-β1-induced bladder smooth muscle cell hypertrophic and fibrotic response: Implication for a role of microRNA in bladder wall remodeling caused by bladder outlet obstruction. Cell. Signal..

[55] Lin C.-H.H., Yu M.-C.C., Tung W.-H.H., Chen T.-T.T., Yu C.-C.C., Weng C.-M.M., Tsai Y.-J.J., Bai K.-J.J., Hong C.-Y.Y., Chien M.-H.H. (2013). Connective tissue growth factor induces collagen I expression in human lung fibroblasts through the Rac1/MLK3/JNK/AP-1 pathway. Biochim. Biophys. Acta Mol. Cell Res..

[56] Wang J., Harris A., Prendes M.A., Alshawa L., Gross J.C., Wentz S.M., Rao A.B., Kim N.J., Synder A., Siesky B. (2017). Targeting Transforming Growth Factor-β Signaling in Primary Open-Angle Glaucoma. J. Glaucoma.

[57] Mietzner R., Breunig M. (2019). Causative glaucoma treatment: Promising targets and delivery systems. Drug Discov. Today.

[58] Tomarev S.I., Wistow G., Raymond V., Dubois S., Malyukova I. (2003). Gene expression profile of the human trabecular meshwork: NEIBank sequence tag analysis. Investig. Ophthalmol. Vis. Sci..

[59] Rao P.V., Pattabiraman P.P., Kopczynski C. (2017). Role of the Rho GTPase/Rho kinase signaling pathway in pathogenesis and treatment of glaucoma: Bench to bedside research. Exp. Eye Res..

[60] Wang S.K., Chang R.T. (2014). An emerging treatment option for glaucoma: Rho kinase inhibitors. Clin. Ophthalmol..

[61] Dallon J.C., Ehrlich H.P. (2008). A review of fibroblast-populated collagen lattices. Wound Repair Regen..

[62] Pattabiraman P.P., Maddala R., Rao P.V. (2014). Regulation of plasticity and fibrogenic activity of trabecular meshwork cells by rho GTPase signaling. J. Cell. Physiol..

[63] Zhang Q., Ye H., Xiang F., Song L.J., Zhou L.L., Cai P.C., Zhang J.C., Yu F., Shi H.Z., Su Y. (2017). miR-18a-5p Inhibits Sub-pleural Pulmonary Fibrosis by Targeting TGF-β Receptor II. Mol. Ther..

[64] Li L., Shi J.Y., Zhu G.Q., Shi B. (2012). MiR-17-92 cluster regulates cell proliferation and collagen synthesis by targeting TGFB pathway in mouse palatal mesenchymal cells. J. Cell. Biochem..

[65] Kolenda T., Guglas K., Kopczyńska M., Sobocińska J., Teresiak A., Bliźniak R., Lamperska K. (2020). Good or not good: Role of miR-18a in cancer biology. Reports Pract. Oncol. Radiother..

[66] Kirwan R.P., Leonard M.O., Murphy M., Clark A.F., O’Brien C.J. (2005). Transforming growth factor-beta-regulated gene transcription and protein expression in human GFAP-negative lamina cribrosa cells. Glia.

[67] Dillinger A.E., Weber G.R., Mayer M., Schneider M., Göppner C., Ohlmann A., Shamonin M., Monkman G.J., Fuchshofer R. (2022). CCN2/CTGF-A Modulator of the Optic Nerve Head Astrocyte. Front. Cell Dev. Biol..

[68] Mahale A., Othman M.W., Al Shahwan S., Al Jadaan I., Owaydha O., Khan Z., Edward D.P. (2015). Altered expression of fibrosis genes in capsules of failed Ahmed glaucoma valve implants. PLoS ONE.

[69] Esson D.W., Neelakantan A., Iyer S.A., Blalock T.D., Balasubramanian L., Grotendorst G.R., Schultz G.S., Sherwood M.B. (2004). Expression of connective tissue growth factor after glaucoma filtration surgery in a rabbit model. Investig. Ophthalmol. Vis. Sci..

[70] Klaassen I., van Geest R.J., Kuiper E.J., van Noorden C.J.F., Schlingemann R.O. (2015). The role of CTGF in diabetic retinopathy. Exp. Eye Res..

[71] Kothary P.C., Badhwar J., Weng C., Del Monte M.A. (2010). Impaired Intracellular Signaling May Allow Up-Regulation of CTGF-Synthesis and Secondary Peri-Retinal Fibrosis in Human Retinal Pigment Epithelial Cells from Patients with Age-Related Macular Degeneration.

[72] He S., Chen Y., Khankan R., Barron E., Burton R., Zhu D.H., Ryan S.J., Oliver N., Hinton D.R. (2008). Connective tissue growth factor as a mediator of intraocular fibrosis. Investig. Ophthalmol. Vis. Sci..

[73] Greene K.M., Stamer W.D., Liu Y. (2022). The role of microRNAs in glaucoma. Exp. Eye Res..

