# Supplementary material for: MiR-18a-5p Targets Connective Tissue Growth Factor Expression and Inhibits Transforming Growth Factor β2-Induced Trabecular Meshwork Cell Contractility
Source: Genes (Basel). 2022 Aug 22;13(8):1500. doi: 10.3390/genes13081500 (PMC9408287; doi:10.3390/genes13081500)
Supplement: Supplementary file 1 [file genes-13-01500-s001.zip › Figure S1 Characterisation of donor TM cells.pdf]

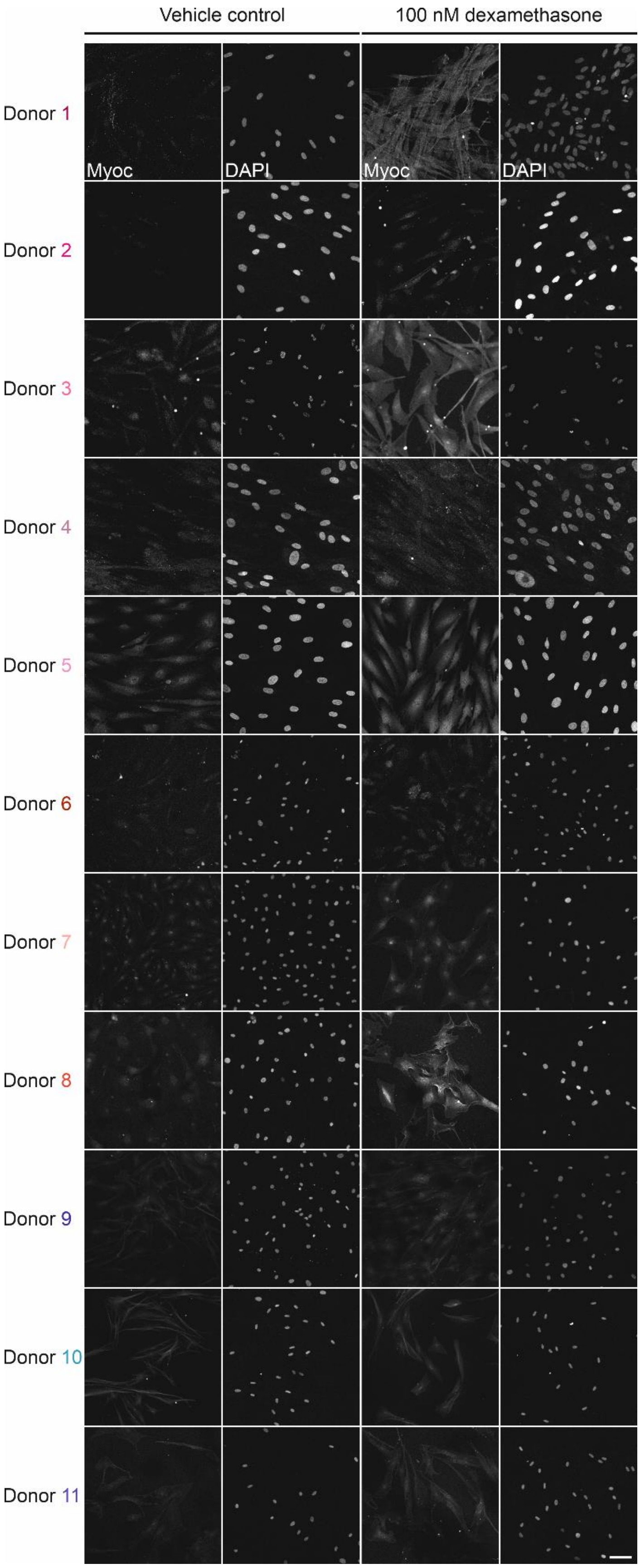

Figure S1 Characterisation of donor TM cells. Indirect immunofluorescent staining for myocilin in TM cells treated and untreated with 100 nM dexamethasone. Scale bar = 50 µm.
