# Supplementary material for: MiR-18a-5p Targets Connective Tissue Growth Factor Expression and Inhibits Transforming Growth Factor β2-Induced Trabecular Meshwork Cell Contractility
Source: Genes (Basel). 2022 Aug 22;13(8):1500. doi: 10.3390/genes13081500 (PMC9408287; doi:10.3390/genes13081500)
Supplement: Supplementary file 1 [file genes-13-01500-s001.zip › Figure S2 Characterisation of TM cells from donors 6 to 11 by western blot for myocilin.pdf]

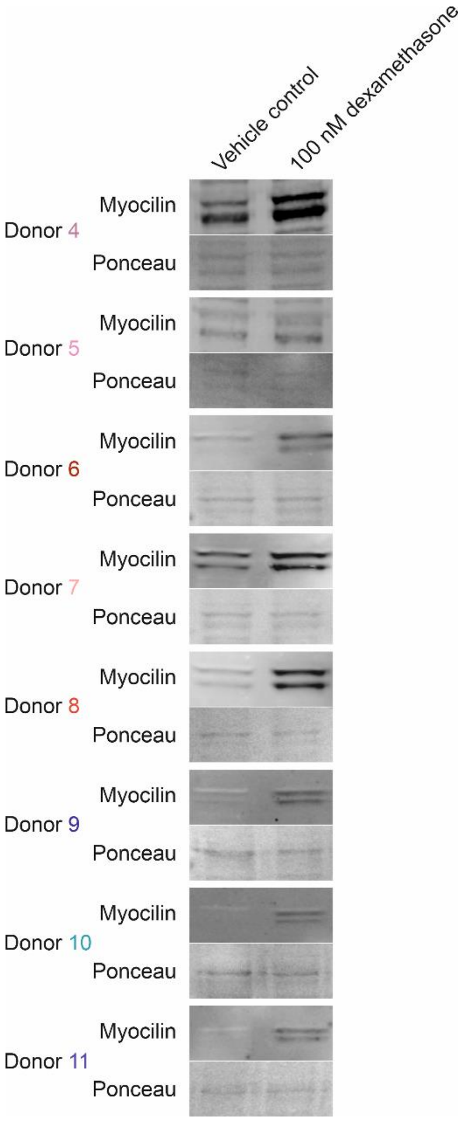

Figure S2 Characterisation of TM cells from donors 4 to 11 by western blot for myocilin. Myocilin expression after 100 nM dexamethasone treatment was measured by western blot. Donors 1, 2 and 3 have been characterised previously[42].
