# Supplementary material for: MiR-18a-5p Targets Connective Tissue Growth Factor Expression and Inhibits Transforming Growth Factor β2-Induced Trabecular Meshwork Cell Contractility
Source: Genes (Basel). 2022 Aug 22;13(8):1500. doi: 10.3390/genes13081500 (PMC9408287; doi:10.3390/genes13081500)
Supplement: Supplementary file 1 [file genes-13-01500-s001.zip › Figure S4 Validation of lentiviral cell lines.pdf]

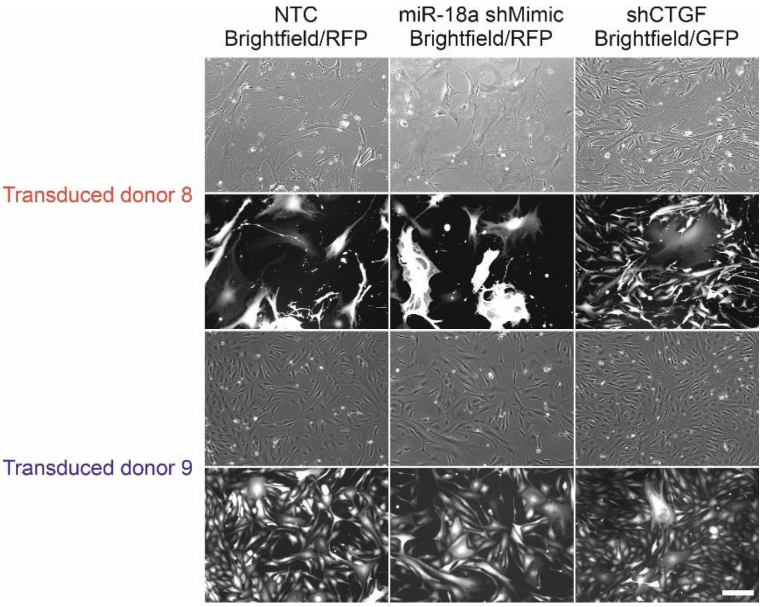


**Figure S4 Validation of lentiviral cell lines.** Live cell brightfield and fluorescence in cells transduced with lentiviruses expressing RFP or GFP. Scale bar = 100µm.
