# Supplementary material for: MiR-18a-5p Targets Connective Tissue Growth Factor Expression and Inhibits Transforming Growth Factor β2-Induced Trabecular Meshwork Cell Contractility
Source: Genes (Basel). 2022 Aug 22;13(8):1500. doi: 10.3390/genes13081500 (PMC9408287; doi:10.3390/genes13081500)
Supplement: Supplementary file 1 [file genes-13-01500-s001.zip › Figure S5 Lentiviral.pdf]

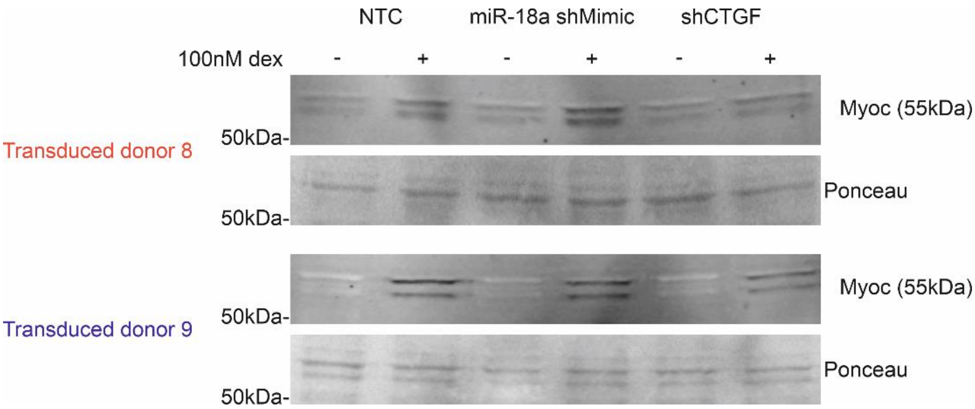

Figure S5 Lentiviral-transduced TM cells display characteristic upregulation of myocilin by dexamethasone. Expression of myocilin in lentivirus transduced cell lines, with and without dexamethasone treatment.
