# Supplementary material for: MiR-18a-5p Targets Connective Tissue Growth Factor Expression and Inhibits Transforming Growth Factor β2-Induced Trabecular Meshwork Cell Contractility
Source: Genes (Basel). 2022 Aug 22;13(8):1500. doi: 10.3390/genes13081500 (PMC9408287; doi:10.3390/genes13081500)
Supplement: Supplementary file 1 [file genes-13-01500-s001.zip › Table S1 Demographic information of human donor rims.pdf]

Table S1 Demographic information of human donor rims

Donor	Age	Sex
1	57	Male
2	65	Male
3	64	Male
4	21	Male
5	88	Female
6	46	Female
7	77	Male
8	43	Female
9	52	Male
10	83	Female
11	68	Male
