# Supplementary material for: MiR-18a-5p Targets Connective Tissue Growth Factor Expression and Inhibits Transforming Growth Factor β2-Induced Trabecular Meshwork Cell Contractility
Source: Genes (Basel). 2022 Aug 22;13(8):1500. doi: 10.3390/genes13081500 (PMC9408287; doi:10.3390/genes13081500)
Supplement: Supplementary file 1 [file genes-13-01500-s001.zip › Table S2 miScript primer assay sequences.pdf]

Table S2 miScript primer assay sequences

miRNA	Probe sequence
hsa-miR-18a-5p (MIMAT0000072)	5'-UAAGGUGCAUCUAGUGCAGAUAG
hsa-miR-19a-3p (MIMAT0000073)	5'-UGUGCAAAUCUAUGCAAAACUGA
hsa-miR-19b-3p (MIMAT0000074)	5'-UGUGCAAAUCCAUGCAAAACUGA
hsa-miR-26a-5p (MIMAT0000082)	5'-UUCAAGUAAUCCAGGAUAGGCU
hsa-miR-26b-5p (MIMAT0000083)	5'-UUCAAGUAAUUCAGGAUAGGU
hsa-miR-133b (MIMAT0000770)	5'-UUUGGUCCCCUUCAACCAGCUA
hsa-miR-199a-5p (MIMAT0000231)	5'-CCCAGUGUUCAGACUACCUGUUC
hsa-miR-199b-5p (MIMAT0000263)	5'-CCCAGUGUUUAGACUAUCUGUUC
SNORD 61	Unavailable; transcript: NR_002735 (73bp)
SNORD 68	Unavailable; transcript: NR_ 002450 (72bp)
