# Supplementary material for: MiR-18a-5p Targets Connective Tissue Growth Factor Expression and Inhibits Transforming Growth Factor β2-Induced Trabecular Meshwork Cell Contractility
Source: Genes (Basel). 2022 Aug 22;13(8):1500. doi: 10.3390/genes13081500 (PMC9408287; doi:10.3390/genes13081500)
Supplement: Supplementary file 1 [file genes-13-01500-s001.zip › Table S3 Unlabelled gene specific human primers.pdf]

Table S3 Unlabelled gene specific human primers

Gene	Sequence	Location (exon)	Amplicon (bp)
CCN2	F: 5'-GCTTACCGACTGGAAGACACG<br>R: 5'-CGGATGCACTTTTTGCCCTT	Exon 3-4<br>Exon 5	233
RPLP0	F: 5'-GCAGCATCTACAACCCTGAAGTGCTTGA<br>R: 5'-GGTAGCCAATCTGCAGACAGACACTGG	Exon 6<br>Exon 7	108
GAPDH	F: 5'-GGAGCGAGATCCCTCCAAAAT<br>R: 5'-GGCTGTTGTCATACTTCTCATGG	Exon 4-5<br>Exon 6	197
