# Supplementary figures and images for: MiR-18a-5p Targets Connective Tissue Growth Factor Expression and Inhibits Transforming Growth Factor β2-Induced Trabecular Meshwork Cell Contractility
Source: Genes (Basel). 2022 Aug 22;13(8):1500. doi: 10.3390/genes13081500 (PMC9408287; doi:10.3390/genes13081500)

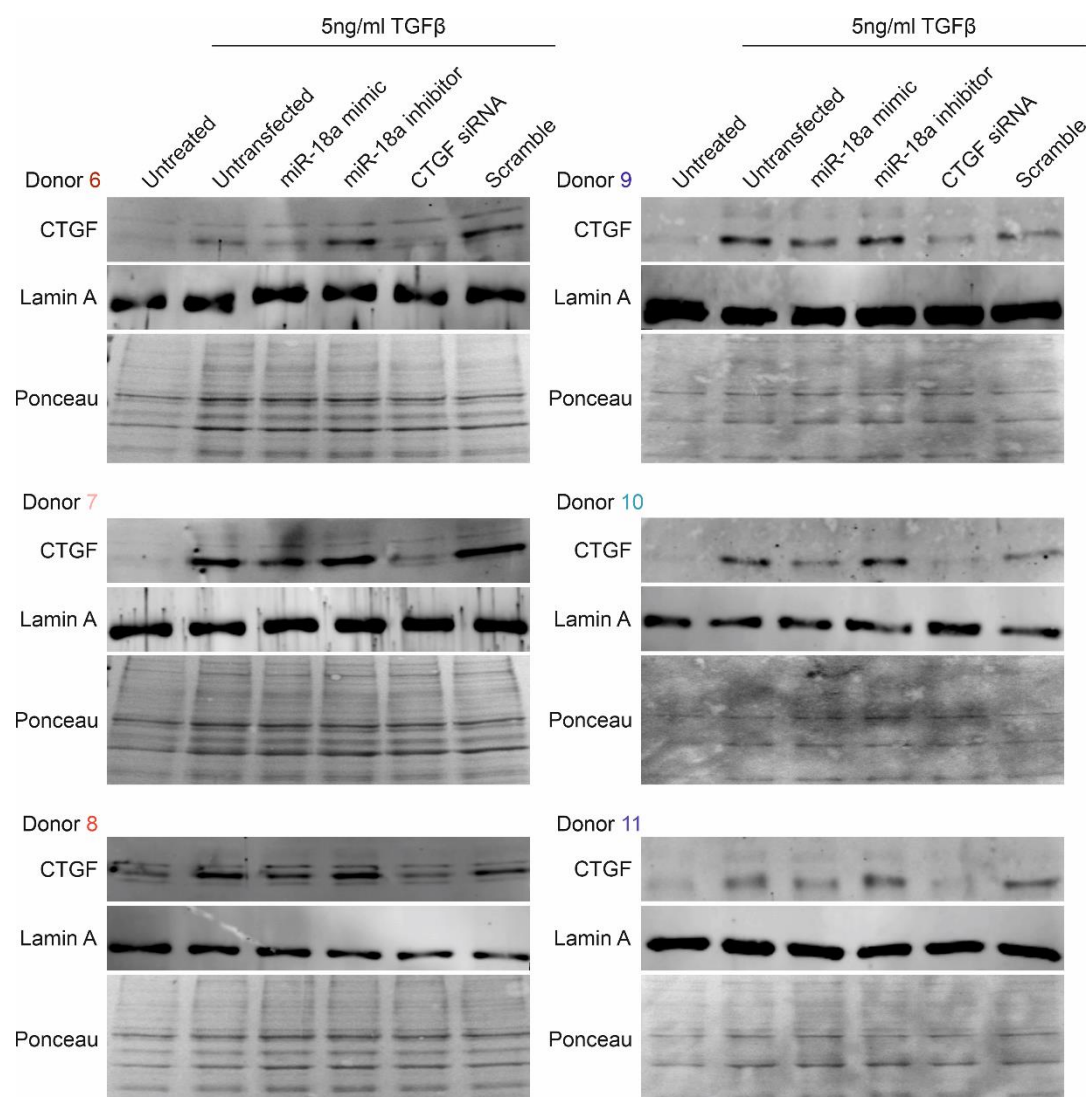

**Figure S3 All western blots for CTGF and Lamin A.**

Supplement: Supplementary file 1 [file genes-13-01500-s001.zip › Figure S3 All western blots for CTGF and Lamin A.pdf]

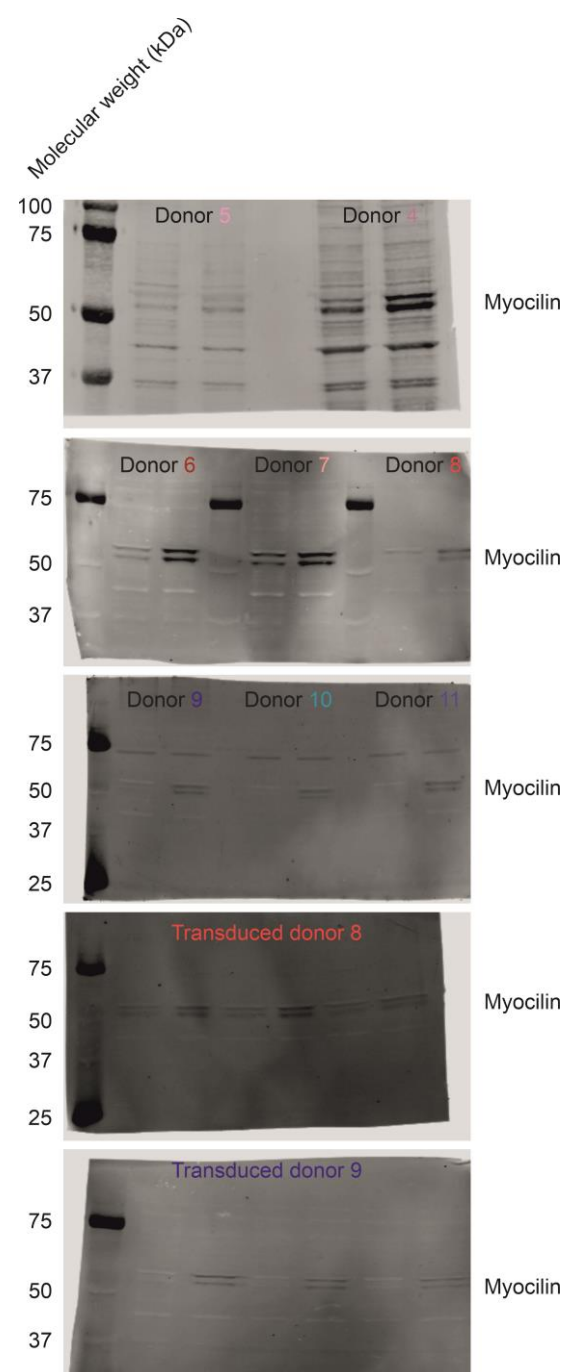

**Figure S6 Original myocilin blot images.**

Supplement: Supplementary file 1 [file genes-13-01500-s001.zip › Figure S6 Original myocilin blot images.pdf]

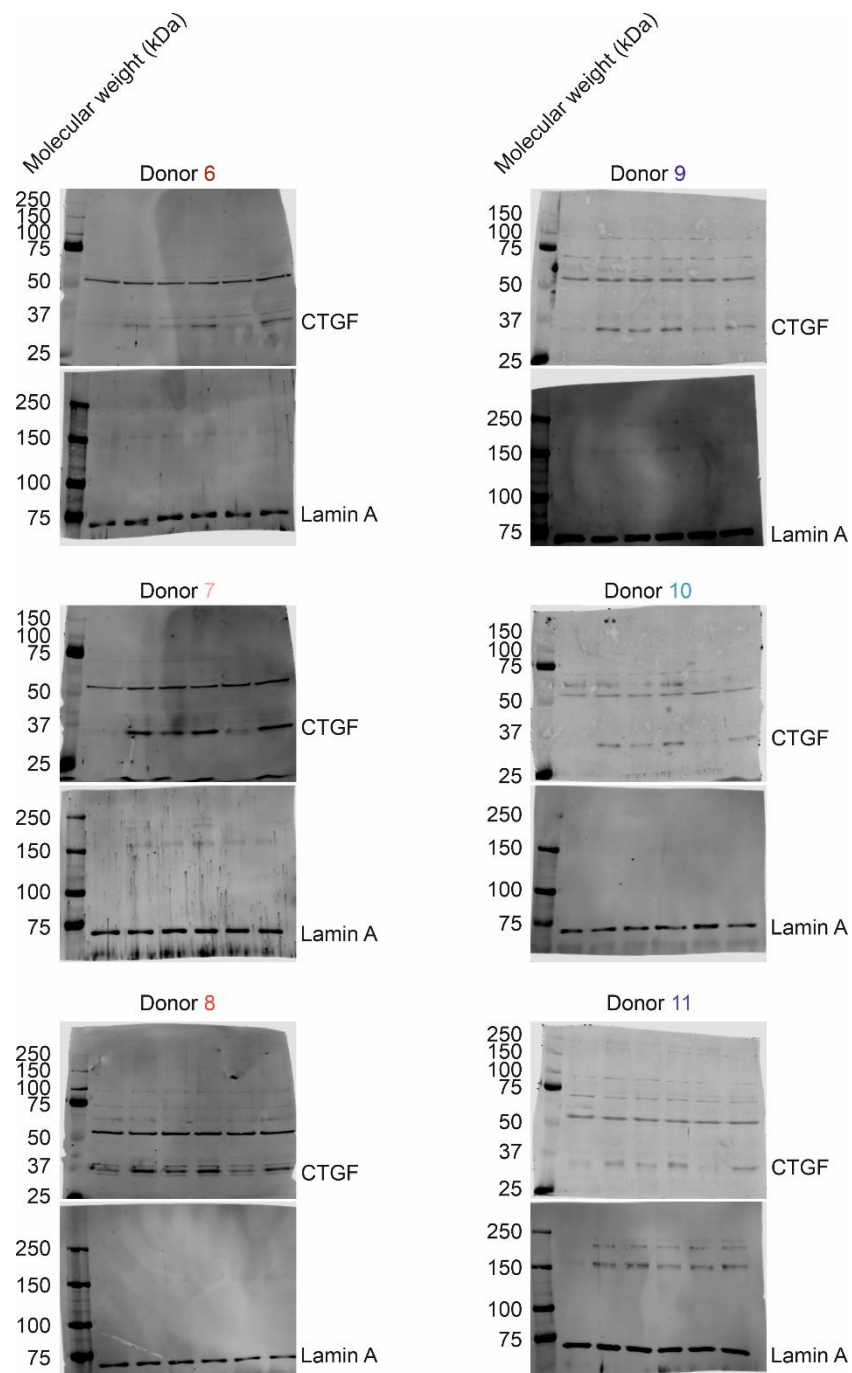

**Figure S7 Original CTGF and Lamin A blot images.**

Supplement: Supplementary file 1 [file genes-13-01500-s001.zip › Figure S7 Original CTGF and Lamin A blot images.pdf]
